# A Deep Learning Algorithm for Classifying Local Material Defects in Aluminum Castings Based on Signals from a Self-Excited Acoustic System SAS

**DOI:** 10.3390/s26216798

**Published:** 2026-10-27

**Authors:** Paweł Gut, Krzysztof Lalik, Ksenia Ostrowska, Paweł Wołkanowski, Ireneusz Dominik

**Affiliations:** 1 Faculty of Mechanical Engineering and Robotics, AGH University of Krakow, Al. Mickiewicza 30, 30-059 Krakow, Poland; pgut@agh.edu.pl (P.G.); dominik@agh.edu.pl (I.D.); 2 Mechanical Faculty, PK Krakow University of Technology, Jana Pawła II 37 Avenue, 31-864 Krakow, Poland; ksenia.ostrowska@pk.edu.pl (K.O.); pawel.wolkanowski@pk.edu.pl (P.W.); 3 Doctoral School of the PK Krakow University of Technology, PK Krakow University of Technology (PK), 24 Warszawska Street, 31-155 Krakow, Poland

**Keywords:** self-excited acoustic system, non-destructive testing, Audio Spectrogram Transformer, deep learning, defect detection

## Abstract

This article presents a deep learning algorithm for classifying local material inhomogeneities in aluminum alloy castings based on signals recorded using a self-excited acoustic system (SAS). The aim of this study is to evaluate the potential of using acoustic signals generated in a self-excited system to distinguish between defect-free specimens and those containing internal material defects. The tests were conducted on two specimens with the same geometry: a reference specimen without defects and a specimen with internal defects confirmed by X-ray inspection. Measurements were taken at six corresponding measurement points. A preliminary frequency analysis showed that the presence of inhomogeneities affects the system’s acoustic response; however, the nature of these changes depends on the measurement location and does not allow for the use of a single universal decision criterion based solely on the dominant frequency. For this reason, a classification procedure was developed using the time-frequency representation of the signal and the Audio Spectrogram Transformer architecture. The procedure includes SAS signal acquisition, preprocessing, signal transformation into spectrograms, feature extraction using the AST model, and classification of samples as OK or NOK. The proposed algorithm was evaluated using accuracy, precision, recall, F1-score, AUC, and a confusion matrix. The results indicate that the proposed method can support the non-destructive testing of aluminum castings and serves as a basis for the further development of acoustic signal classification algorithms for the detection of localized material defects.

## 1. Introduction

Aluminum alloy castings are widely used in the automotive sector because of their favorable strength-to-weight ratio. Their quality can, however, be compromised by defects formed during casting, mainly porosity (voids, gas bubbles) and non-metallic inclusions (e.g., oxides, slag). Such defects cause localized stress concentrations and structural weakening, which may reduce mechanical properties or lead to premature failure [1], so it is crucial to detect them already at the production stage. Studies using X-ray tomography and metallography indicate that the size, morphology, and location of pores affect local stress concentrations and fatigue life [2], and computed tomography (CT) enables three-dimensional characterization of the distribution of internal porosity [3]. Nourian-Avval and Fatemi [4] compared metallographic and micro-CT measurements of porosity in high-pressure die-cast aluminum, and Weidt et al. [5] related porosity volume fraction, characteristic pore diameters, and pore morphology in CT data of aluminum castings; porosity assessment thus requires descriptors of both defect size and population structure. The mechanical significance of such descriptors is supported by defect-based fatigue models, which relate crack-initiating defects to the fatigue strength and lifetime of cast aluminum–silicon alloys [6,7]. These works also separate the detection of a material anomaly from the assessment of its structural criticality: a screening classifier can flag anomalies, whereas predicting residual lifetime requires additional evidence linking its output to defect geometry, loading, and fracture-mechanical parameters. Whether CT-derived pore descriptors map onto acoustic features must be established experimentally.

Quality control of aluminum castings is currently dominated by radiography (X-ray) and CT. These methods image the internal structure but are relatively slow, costly, and require specialized equipment, which makes 100% inspection on the production line difficult [8]: a typical CT cycle for a single casting takes several minutes, whereas the production cycle takes several dozen seconds [8]. Moreover, even a thorough analysis of X-ray images can overlook minor inclusions or low-contrast porosity [1]. Deep learning accelerates the interpretation of radiographs: models detect minor defects in X-ray images of aluminum castings [9], detect and classify defects in die-castings [10], have been analyzed on industrial radiographic datasets [11], and are combined with synthetic data for porosity characterization [12]. Parlak and Emel [13] went further and assigned grades to internal defects in high-pressure aluminum castings through segmentation and classification standards, showing that image-based learning can be coupled with an explicit quality assessment. This is also a relevant reference for acoustic classification: the diagnostic value of a learned representation depends on its correspondence with a defined inspection target. Faster interpretation of an acquired radiograph does not, however, shorten its acquisition. This paper therefore examines a different diagnostic channel, the local acoustic response of the casting, and its subject is the signals of a self-excited acoustic system (SAS), described below.

The SAS belongs to the resonance methods, in which vibrations are excited in a sample and the resulting natural frequencies are analyzed—an approach commonly referred to as Acoustic Resonance Technology (ART) [14]. Because natural frequencies depend on the distribution of mass and stiffness, a defect (a crack, a void, or a local inhomogeneity) changes the resonance spectrum. ART can test the entire volume of a component within seconds, is easy to automate, and requires relatively inexpensive equipment. For aluminum castings, it enables rapid sorting into good and defective parts [14]; Lechner et al. [15] combined resonance vibrations with FEM analysis to locate cavities, noting the influence of defect location and manufacturing variation, and Heinrich et al. [16] showed that the training data must represent the natural variability of the geometry and properties of the examined parts.

The SAS uses positive self-coupling to induce self-sustaining natural vibrations in the tested object [17]. A vibration exciter (e.g., a loudspeaker or other vibrating actuator) drives the object, a sensor (e.g., a piezoelectric accelerometer) detects the vibration passing through the structure, and the amplified signal is fed back to the exciter, closing the positive feedback loop. With sufficient gain, the system enters self-excited oscillation: the specimen vibrates at one of its natural frequencies (the so-called system boundary cycle) without external continuous excitation, analogously to the acoustic coupling between a microphone and a loudspeaker.

Several self-excitation frequencies are usually possible, so the system can select different vibration modes, which causes sudden frequency jumps and complicates the analysis. Limiting the frequency band of the feedback loop by filtering “locks” the system to a selected range and a preferred mode. Dominik et al. [18] proposed preliminary signal filtering in the SAS system, which stabilized the obtained frequencies and facilitated the correlation of frequency changes with stress changes; the solution was subsequently developed into an intelligent digital filter implemented on an FPGA platform, which eliminated unwanted frequency jumps in mining anchor tests. Lalik et al. [17] showed that, with the bandwidth limited to 600–1000 Hz, the vibration frequencies of the anchors became stable in field tests, so that a potentially damaged anchor whose frequency deviated significantly from the others could be identified.

Interpreting resonance measurements is, however, not trivial. Natural frequencies depend not only on defects but also on permissible dimensional deviations, variations in material properties, and boundary conditions during measurement; these effects overlap and shift individual frequencies, so a reliable assessment based solely on natural frequencies requires advanced analysis, specifically the solution of an inverse problem. Perturbation methods and inverse problems have been used to relate frequency shifts to the nature and location of a defect [19,20], but the variation resulting from acceptable manufacturing tolerances may overlap with the shifts caused by an actual defect, which makes a single simple frequency criterion difficult to apply [21,22]. Heinrich et al. [14] classified parts as good or defective from measured natural frequencies, using simulation data to account for geometric variance, and observed that minor defects may remain undetected when the frequency variation caused by dimensional tolerances is comparable to the effect of the defect.

Machine learning on multidimensional signal features therefore plays an increasingly important role. Ju et al. [23] used time-frequency features and one- and two-class classification in the ART of sintered gears, and Kraljevski et al. [24] compared classical and deep learning models in the acoustic inspection of glass bottles using a small and unbalanced dataset. A review of deep learning in nondestructive evaluation notes that automatic feature extraction can increase the level of automation but requires representative data and clearly defined validation [25]. Related work on anomalous sound detection in industrial machinery [26] and on acoustic leak detection in pipelines, using enhanced continuous-wavelet-transform scalograms with CNN-based feature extraction and ANN classification [27] or time- and frequency-domain acoustic-emission features with several supervised classifiers [28], concerns different physical systems but shows that diagnostically relevant information can be distributed across time-frequency and multi-feature representations rather than captured by a single scalar descriptor.

Learned acoustic representations have been used in both material inspection and machinery monitoring. Jarreau et al. [29] used a convolutional neural network to classify anomalies in a metallic medium from the amplitude and phase of transmitted acoustic signals measured at different lateral positions (spatially resolved acoustic measurements can thus contain discriminative information on the position and material of an anomaly), and Müller et al. [30] used features of networks pretrained for image classification for unsupervised anomaly detection in machine sounds, outperforming convolutional-autoencoder approaches in their setting. In bearing diagnostics, Neupane and Seok [31] reviewed deep-learning approaches on the Case Western Reserve University dataset, and Orhan et al. [32] compared short-time Fourier transform, continuous wavelet transform, Hilbert–Huang transform, and Wigner–Ville representations with a pretrained vision transformer, showing that classification performance depends on the interaction between the representation and the diagnostic task. These results support the use of transfer learning and multidimensional representations in acoustic diagnostics, but they concern transmitted waves, passive machine sounds, and rotating machinery. None of them establishes performance for a self-excited feedback system, in which the specimen response and the excitation dynamics are coupled, or for casting inspection.

The literature therefore does not resolve whether the full representation of a SAS signal allows the local state of the material in a casting to be classified without an individual selection of the filtering band for each measurement point. Answering this question also requires independent data for evaluation. Data leakage is a recurrent cause of overoptimistic conclusions in machine-learning-based science [33], and in bearing diagnostics segment-wise and condition-wise partitioning has been shown to introduce spurious correlations, which bearing-wise separation avoids [34]. For acoustic casting inspection, signal segments, recordings, and specimens must be distinguished: keeping segments of one recording in a single partition removes one source of dependence, but recordings of the same casting may still share specimen-specific characteristics. With two physically matched castings, the present evaluation therefore addresses discrimination within the investigated specimens, not generalization to a production population (Section 4.2).

The objective of this study is to develop and evaluate a deep learning algorithm for classifying local material defects in aluminum castings based on SAS signals. In the first stage, the frequency response of the system was analyzed in measurements without filtering and with bandpass filtering of the feedback loop, to assess whether the dominant self-excitation frequency could serve as a simple diagnostic feature and whether a single filtering band suitable for all examined locations could be selected. In the second stage, given the limitations of the frequency-domain approach, the full SAS signals from the unfiltered measurements were transformed into a time-frequency representation, and the Audio Spectrogram Transformer (AST) architecture was used to classify the samples as OK and NOK. This combines classical frequency analysis and SAS loop filtering with a multidimensional signal representation. To contextualize AST against compact deep-learning alternatives, SqueezeNet, MobileNet, YOLOv8-nano, and ShuffleNetV2 x0.5 were additionally evaluated on the same log-mel representation and recording-level splits.

The novelty of this work is threefold and rests on the quantitative evidence presented in Section 3 and Section 4:

1. Evidence that single-frequency criteria are insufficient within one part. Unlike prior ART studies that rely on simulation-augmented frequency features [14] or FEM-assisted localization [15], we show experimentally, on two physically matched castings measured at six corresponding points, that neither the dominant self-excitation frequency nor any single feedback-loop filtering band (600–1600, 1600–2600, and 2600–3600 Hz) provides a consistent diagnostic criterion across the six measurement locations; the $f_{0}$-only baseline achieves near-chance accuracy (0.4733±0.0315). To our knowledge, this is a direct quantitative demonstration of the failure of single-frequency criteria at the within-part level, as opposed to the variation across parts caused by dimensional tolerances reported previously [14]. The experiment documents this failure; it does not by itself identify its physical mechanism.
2. Cross-domain transfer of the Audio Spectrogram Transformer to SAS signals. We apply the AST, pretrained on a general-purpose audio corpus [35] with no relation to structural vibration, to SAS-based defect classification. To our knowledge, this cross-domain transfer has not been demonstrated previously for self-excited acoustic diagnostics of castings. Within the investigated dataset, it dispenses with the manual, per-point band selection that our frequency-domain experiments (Section 3) show to be needed but not sufficient for a frequency-only approach.
3. Fully robotized multi-point measurement. The SAS head is positioned by an industrial robot arm across all six points with a fixed contact force and a total dwell time of approximately 2 s per point (1 s to reach a stable self-excited limit cycle, plus 1 s of acquisition). This configuration demonstrates the technical feasibility of automated multi-point inspection, in contrast to the CT-based workflows whose multi-minute cycle times per part make 100% inline coverage difficult [8] and to the manually operated setups of the ART studies cited above.

## 2. Research Methodology

The tests were conducted on two aluminum alloy castings with identical geometries. These were passenger car suspension components cast from aluminum alloy (Figure 1). One of the samples contained intentionally introduced internal defects (an “NOK” sample—not OK), while the other was free of defects (an “OK” sample). The defects in the NOK sample had been previously identified using industrial X-ray radiography; these were primarily clusters of non-metallic inclusions and shrinkage porosity in specific areas of the casting. Complementary computed tomography of the NOK casting confirmed distributed internal void-type porosity; across the analyzed volume, the detected indications exhibited equivalent diameters of approximately 0.36–2.41 mm, while local porosity hot spots reached maximum porosity values of up to 10%.

Based on the X-ray results (Figure 2), six measurement points were selected on the casting’s surface, corresponding to the locations of the diagnosed material discontinuities. The same locations were marked on the OK sample, allowing for a comparison of the acoustic response of the defective and defect-free material at corresponding locations. The six SAS measurement locations were selected from areas showing the most pronounced porosity indications identified in the radiographic and CT assessment.

### 2.1. X-Ray CT Characterization of the Castings

To characterize the internal defects in more detail, X-ray computed tomography (CT) was performed on six castings, designated S1–S6, using a coordinate-measuring industrial computed tomograph, Waygate Technologies Phoenix V|tome|x M240 Metrology Edition. Each scan consisted of 3500 projections, and every projection was averaged from five exposures of 100 ms each. The tube voltage was 190 kV and the tube current 310 μA. The voxel size during scanning was approximately 150 μm, which corresponds to a single-voxel volume of about 0.0034 mm^3^. Closed-air pores were identified in the VGSTUDIO MAX software from changes in gray value. For each indication, the volume *V*, the surface area *A*, the sphericity, and the diameter of the circle circumscribed on the pore, which characterizes its largest linear dimension, were obtained. The sphericity was defined as in Equation (1).

(1) $$\Psi =\frac{\pi^{1/3}\;{\left(6V\right)}^{2/3}}{A}.$$

In addition, to assess pore size independently of shape, the equivalent spherical diameter and the total porosity, defined as the ratio of the summed volume of all detected pores to the volume of the casting, were computed as in Equation (2).

(2) $$d_{\mathrm{eq}}={\left(\frac{6V}{\pi }\right)}^{1/3}$$

The volumes of castings S1–S6 were 818.1, 756.3, 346.9, 809.3, 935.4, and 754.2 cm^3^, respectively. The indications were not filtered by the Probability parameter, which expresses the probability that an indication is a real material discontinuity, because it increases as the pore shape approaches a sphere, so thresholding would have excluded irregular pores.

With a voxel size of about 150 μm, the detectability of pores is not limited to structures larger than a voxel. Owing to the partial volume effect and subvoxeling, even a material loss smaller than a voxel changes its gray value and can be registered as a pore, because a single voxel can contain both material and void. At the same time, the volume and shape of the smallest indications, comprising only a few voxels, were determined with greater uncertainty; therefore, the conclusions on defect type and severity are based primarily on larger pores.

The range of registered pores differs between the castings. In castings S1–S3, the smallest included pores had a volume of 0.015–0.024 mm^3^, i.e., about 5–7 voxels. In castings S4–S6, indications smaller than a single voxel (from 0.001 mm^3^) were also registered; they made up 8–14% of all indications. In addition, the sphericity in S1–S3 does not exceed 0.81, whereas in S4–S6 it reaches 0.97, so sphericity values were compared only within these two groups of castings.

The CT analysis confirmed closed-air pores in all six castings, located mainly in the pressure die-casting injection regions of the aluminum alloy. Figure 3 shows an example injection region. The largest defects form a chain of elongated, partly coalesced pores extending along the lower part of the casting, and a flat, plate-like pore lies perpendicular to the axis of the part. These large pores are surrounded by a cloud of small, mostly compact pores. This example illustrates that porosity is not distributed uniformly but is concentrated in the injection region.

The number of detected pores ranged from 150 to 2629 per casting (Table 1), and the total porosity was 0.012–0.071%. The pore size distribution is strongly right-skewed. On linear axes (Figure 4), almost all pores of casting S1 cluster near the abscissa: their volume is below 1 mm^3^, and 90% of them have a sphericity between 0.46 and 0.72. Pores of lower sphericity are larger, and the four largest pores stand out from the population: 24.1 mm^3^ at Ψ≈0.32, 7.7 mm^3^ at Ψ≈0.32, 5.1 mm^3^ at Ψ≈0.25, and 4.9 mm^3^ at Ψ≈0.49.

The median pore volume was only 0.021–0.094 mm^3^ across the castings, the 99th percentile was 0.68–6.6 mm^3^, and the largest individual pores reached 2.1–70.5 mm^3^, corresponding to equivalent spherical diameters of 1.6–5.1 mm. The largest diameter of a circle circumscribed on a pore was 6.1–26.7 mm. Because of the strong skewness of the distribution, the arithmetic mean volume is dominated by a few extreme values and does not describe the population reliably; therefore, medians and percentiles are reported (Table 1).

Defect type depends on pore size and differs between the castings (Figure 5). Small pores (V<0.1 mm^3^) were compact, with a mean sphericity of 0.61–0.77 across the castings, which is characteristic of gas porosity. Pores of at least 1 mm^3^ were less compact, with a median sphericity of 0.37–0.67, depending on the casting. In castings S1–S3, the sphericity decreased monotonically with volume (Spearman correlation coefficient ρ from −0.65 to −0.49): the median sphericity falls from about 0.65 for pores of 0.01–0.03 mm^3^ to 0.32–0.39 for pores larger than 3 mm^3^, and 67–100% of the pores larger than 1 mm^3^ had Ψ<0.5, which is typical of shrinkage porosity or coalesced gas pores. In castings S4 and S6, the five largest pores were less irregular (Ψ=0.36–0.68 and 0.55–0.63, respectively). In casting S5, whose largest pore was only 2.1 mm^3^, the largest pores were nearly spherical (Ψ=0.74–0.98), i.e., of gas-porosity character.

The scatter in castings S4–S6 (lower row of Figure 5) is much wider than in S1–S3 and extends down to 0.001 mm^3^. In the size classes from 0.01 to 0.3 mm^3^, the median sphericity is 0.78–0.87, i.e., the pores are mostly compact, whereas pores larger than 1 mm^3^ spread over a wide range of sphericity (Ψ=0.28–0.96 in S4, 0.53–0.98 in S5, and 0.36–0.93 in S6). Pores smaller than 0.01 mm^3^ (about three voxels) have lower sphericity than the next size class, which probably results from the limited resolution rather than from real morphology. Casting S4 contains the two largest pores of the whole data set (70.5 and 44.5 mm^3^, Ψ=0.36 and 0.51). In casting S6, twelve pores larger than 3 mm^3^ (up to 7.5 mm^3^) are clearly separated from the rest of the population, and only one pore lies between 1 and 3 mm^3^.

The size distributions are shown in Figure 6a. The median equivalent diameter is similar in all castings (0.34–0.56 mm), and the 99th percentile lies between 1.1 and 2.3 mm. The curves for S1–S3 start at about 0.31–0.36 mm (2–2.4 voxels), and those for S4–S6 at about 0.12–0.13 mm (below one voxel), which reflects the difference in the range of registered pores described above.

The severity of the defects is governed by a small number of large pores rather than by the mass of micropores. The total porosity is low (0.012–0.071%), and the number of pores of at least 1 mm^3^ ranged from 9 to 101 per casting. As Figure 6b shows, the ten largest pores carried 7–65% of the summed pore volume. The single largest pore accounts for 24% of this volume in casting S1 (24.1 of 101 mm^3^) but only 1% in casting S5. Such large, irregular pores in the injection regions can be initiation sites for fatigue cracks and leakage paths, so they determine the quality assessment of the casting.

### 2.2. SAS Measurement System and Procedure

The measurements were performed using the SAS self-excited acoustic system developed in the laboratory. A schematic diagram of the system is shown in Figure 7. It consisted of a vibration transducer (exciter) in the form of a miniature piezoelectric speaker pressed against the surface of the sample, and a vibration sensor—a piezoelectric accelerometer—that detected the sample’s vibrations at the point of contact.

The signal from the accelerometer was conditioned and amplified, then fed to the exciter’s input, closing the feedback loop. The entire system was integrated with the arm of an industrial robot, at the end of which a holder with a SAS head containing the exciter and sensor was mounted (Figure 8). The use of the robot ensured high repeatability in the positioning of the measuring head and a consistent contact force. These factors are essential for maintaining constant measurement boundary conditions. The robot also enabled the automation of the measurement sequence; the head sequentially touched the designated 6 points on the casting, performing a series of measurements without operator intervention.

Before beginning the measurements, the acoustic system was calibrated and the proper operation of the feedback loop was verified. The amplifier gain was set slightly above the system’s self-excitation threshold so that the oscillations remained stable without saturating the signal. For each measurement point, the procedure was as follows: the SAS transducer was positioned at a specified point on the specimen, and contact with the material initiated self-excitation. After waiting 1 s, once the system had reached a stable amplitude and vibration frequency, the signal from the accelerometer was recorded for a period of 1 s.

The signal was analyzed using a Fourier transform to determine the dominant vibration frequency. The SAS system generated a signal similar to a sine wave, with minor higher-harmonic components; therefore, the frequency of the main peak in the signal’s power spectrum was taken as the dominant frequency $f_{0}$ [1]. In the series used for the direct presentation of frequency results, each point was tested five times, recording separate excitations on both samples.

After performing a series of measurements without filtering—that is, in the full feedback bandwidth—tests were conducted with a frequency band limit in the loop. For this purpose, a 4th-order Butterworth digital bandpass filter, implemented in the FPGA controlling the SAS, was used in the feedback signal path. Three frequency ranges were tested sequentially: 600–1600 Hz, 1600–2600 Hz, and 2600–3600 Hz. The ranges were selected based on preliminary full-band measurement results, in which the observed self-excitation frequencies ranged mainly from several hundred hertz to approximately 3500 Hz. The goal of this stage was not only to limit frequency jumps but also to verify whether there is a single feedback loop band in which a diagnostic response can be stably excited for all six points and various local material discontinuities. For each filter configuration, measurements were repeated at all six points on both castings, analogous to the full-band measurements.

During the measurements, frequency stability over time was also monitored. In the absence of filtering, sporadic jumps in the self-excitation frequency occurred, particularly at points where the accelerometer signal was relatively weak. The use of bandpass filters significantly reduced this effect and stabilized the oscillation within the specified range. At the same time, for some points, the bandwidth limitation resulted in the absence of self-excitation or required an increase in gain. This means that the filter improves stability only when it covers a mode that can be excited at a given location. If the selected band does not cover the appropriate modal range, the loop does not meet the conditions for self-excitation. Already at the methodological stage, this indicated that selecting a single fixed band for the entire part might be insufficient, since the local response depends on the measurement point and the nature of the inhomogeneities.

### 2.3. Framework for SAS Signal Analysis Using AST

Frequency analysis constitutes the first step in the proposed diagnostic procedure. Its purpose is to determine to what extent a single feature—in the form of a dominant frequency—allows for the differentiation between OK and NOK samples. Measurements have shown that the SAS signal responds to changes in the material’s state; however, the frequency value depends on the location, the excited mode, and the loop operating conditions. Additionally, experiments with filtering showed that a frequency band stable at one point does not necessarily allow for self-excitation at another. For this reason, neither a single frequency nor a single global filtering band provides a sufficiently universal basis for a diagnostic decision. This served as the direct justification for moving to an analysis of the full time-frequency representation.

The developed framework (Figure 9) comprises five consecutive stages: acquisition of a stable segment of the SAS signal, preliminary signal preparation, transformation to a time-frequency representation, analysis using the Audio Spectrogram Transformer model, and binary OK/NOK classification. In the section utilizing the AST, only recordings made without bandpass filtering of the feedback loop were analyzed. This allows us to separate the experiment concerning the selection of the SAS operating band from the classification experiment and to assess whether the model can utilize the information present in the full signal without manual selection of the frequency range.

The Audio Spectrogram Transformer is an architecture derived from the Vision Transformer concept [36], adapted for the analysis of time-frequency representations of audio signals [37]. The spectrogram is divided into smaller segments (patches), which are then analyzed using a self-attention mechanism. This allows the model to account for long-range dependencies simultaneously along the time and frequency axes, which is particularly important in the analysis of SAS signals, where the effect of a defect may manifest not only through a change in the dominant frequency but also through a change in the energy distribution, the presence of side components, and a change in the stability of oscillations. The development of models trained on spectrogram representations includes, among others, SSAST [38], PaSST [39], and BEATs [40], while research on PANNs [41] has confirmed the usefulness of transfer learning from large audio datasets. The AST checkpoint used in this work was pretrained on AudioSet, a large dataset of annotated sound events [35].

### 2.4. Data Collection and Preparation of the Time-Frequency Representation

The dataset used in the AST section was created based on two physical castings: one OK and one NOK, examined at six corresponding points. At each point, 10 one-second recordings were made, and the full measurement sequence was repeated ten times. This yielded 100 recordings per point for each casting, for a total of 1200 signals—600 for each class. All data used for training were derived from measurements without SAS loop bandpass filtering. The sampling frequency was 10 kHz; therefore, a single recording contained 10,000 samples.

Preprocessing began by standardizing the signal length to 10,000 samples through truncation or padding with zeros, followed by z-score normalization performed separately for each recording. The time-frequency representation was determined as a mel-frequency power spectrogram with parameters n_fft = 1024, hop_length = 256, n_mels = 128, and a frequency range of 0–4800 Hz. After limiting the minimum value to $10^{-8}$, a logarithmic transformation (ln) was applied, after which each spectrogram was normalized again using the z-score method and the standard deviation was scaled by a factor of 0.5. The final input tensor had dimensions of 40×128 (time frames × mel bands). In the ML track, no filtering corresponding to the experimentally tested bands or data augmentation was applied.

These preprocessing parameters were fixed before model evaluation. At a sampling rate of 10 kHz, $n_{\mathrm{fft}}=1024$ corresponds to a Fourier-bin spacing of approximately 9.8 Hz, while hop_length = 256 gives a frame advance of 25.6 ms, providing a compromise between spectral detail and temporal tracking over the 1 s recording. The 128 mel bands reduce input dimensionality while retaining finer resolution at lower frequencies, and the upper limit of 4800 Hz remains below the 5 kHz Nyquist frequency. The mel representation was selected primarily because the pretrained AST was developed for log-mel inputs, thereby enabling transfer learning from AudioSet [35,37]; it is not assumed to be intrinsically superior to a linear-frequency representation for structural vibration. The 0.5 factor is a fixed numerical scaling applied after z-score normalization to control input variance; it was held constant for all samples and seeds and was not tuned on the validation or test sets. A direct mel-versus-linear spectrogram ablation was outside the scope of the present study.

### 2.5. Model Configurations and Training Procedure

For classification, we used ASTForAudioClassification from the Hugging Face Transformers library, initialized with the MIT/ast-finetuned-audioset-10-10-0.4593 checkpoint, which had previously been trained on the AudioSet dataset. The classification head was adapted to two classes, OK/NOK, and the entire backbone was left trainable; thus, full fine-tuning of all 85.3 million parameters was performed. Training was performed in the PyTorch 2.5.1/Python 3.11.14/Hugging Face environment on a CUDA 12.1 device using the AdamW optimizer, with a learning rate of $1\times 10^{-4}$, weight decay of $1\times 10^{-4}$, a batch size of 8, and the CosineAnnealingLR schedule. The maximum number of epochs was 50; the best checkpoint was selected based on F1 on the validation set, and early stopping was set to patience = 3. The stability of the procedure was assessed across five runs for seeds 88, 14, 53, 34, and 72.

The recorded AST training histories were additionally examined to assess epoch-wise overfitting. In several runs, training loss continued to decrease after validation F1 had reached its maximum, while validation loss plateaued or increased. This behavior indicates a tendency toward within-dataset overfitting and supports the use of best-checkpoint selection by validation F1 together with early stopping. These controls limit overfitting across epochs, but because the training and validation recordings originate from the same two physical castings, they do not address casting-level overfitting or establish generalization to unseen parts.

Four compact CNN baselines were included: SqueezeNet, MobileNet, YOLOv8-nano, and ShuffleNetV2 x0.5 [42]. All used the same log-mel inputs and splits as AST. For ShuffleNetV2, ImageNet-pretrained weights were used its first convolution was adapted from three input channels to one by averaging the pretrained RGB kernels, and the final classifier was replaced by a two-output layer. No resizing to 224 × 224 or ImageNet RGB normalization was applied; the network received the native 1 × 128 × 40 representation and contained 343,410 trainable parameters. Training used AdamW (learning rate 1 × 10^−4^, weight decay 1 × 10^−4^), batch size 8, cosine annealing for a maximum of 50 epochs, best-checkpoint selection by validation F1, and early stopping with patience = 3.

### 2.6. Validation Strategy and Baseline Models

The dataset was stratified into train/validation/test sets in a 70/15/15 ratio at the file level, which corresponded to 840, 180, and 180 records, respectively. To provide a fair comparison with classical frequency analysis, a baseline model was constructed using only the dominant frequency $f_{0}$ determined from the FFT maximum. Logistic regression was trained on this single feature using the same data splits as for AST. This comparison allows for a direct assessment of whether the time-frequency representation provides diagnostic information beyond the location of the dominant spectral maximum. The deep-learning reference set comprised SqueezeNet, MobileNet, YOLOv8-nano, and ShuffleNetV2 x0.5. These models used the same unfiltered recordings, log-mel representation, and five train/validation/test splits as AST, allowing the transformer to be compared with both a scalar frequency baseline and several compact convolutional alternatives.

It should be noted that, since two physical castings were available, splitting the data at the file level is not equivalent to validation on new, previously unseen castings. Repeats from the same casting may occur in different parts of the dataset. Therefore, the results should be interpreted as an assessment of the ability to classify recordings within the experimental setup under study, rather than as definitive proof of generalizability to a production population of castings. Because repeated measurements from the same physical casting can occur in both the training and test subsets, the recording-level split may provide an optimistic estimate of performance compared with an evaluation on fully independent castings. Accordingly, the reported metrics quantify OK/NOK discrimination within the present two-casting experiment and should not be interpreted as an estimate of classification performance on unseen castings.

### 2.7. Interpretability Analysis

To provide a qualitative interpretation of the AST decisions, a gradient-based saliency analysis was performed for the representative model trained with seed = 53, i.e., the same run used for the confusion matrix. For each correctly classified test recording, the absolute gradient of the predicted-class logit with respect to the input log-mel spectrogram was calculated. The resulting saliency maps were averaged separately for the OK and NOK classes (n = 87 and n = 88, respectively). The two class-averaged maps were jointly normalized to the range [0, 1] using a common maximum so that relative gradient magnitudes could be visualized on the same scale. Only the time-frequency region covered by the AST patch embedding was displayed.

## 3. Results

Measurements of self-excited vibration frequencies at six points on both specimens (OK and NOK) across the full range of the feedback loop are summarized in Table 2. For each point, the average measured cycle frequency is given along with the standard deviation. Figure 10 shows the raw frequency values for successive recordings. The results reveal clear differences between the responses of the OK and NOK castings; however, the magnitude and direction of the frequency shift depend on the measurement point. For this reason, the $f_{0}$ value alone does not constitute a global, monotonic diagnostic criterion.

The values presented in Table 2 confirm that the differences between the OK and NOK samples are greater than the repeatability spread; however, their direction and magnitude depend heavily on location. This means that the dominant frequency $f_{0}$ is sensitive to changes in the dynamic response of the casting, but it is not, in itself, a universal indicator of the presence of a defect.

The dependence of the response on the measurement point can be attributed to the local geometry, the distribution of mass and stiffness, and the vibration mode selected by the self-excited system. Consequently, the presence of inhomogeneities does not necessarily lead to a uniform change in $f_{0}$ at every location, and with small frequency shifts, the use of a simple decision threshold becomes ambiguous.

In a subsequent experiment, it was investigated whether limiting the feedback loop to a selected band could unify the system’s response and facilitate the interpretation of measurements. Filtering stabilized the self-excitation within the selected range, but its effectiveness depended on the measurement point.

As shown in Figure 11, the 600–1600 Hz band allowed for stable operation only at points 1 and 4, while higher frequency ranges encompassed different sets of local modes. Thus, a single filter setting suitable for all points of the tested casting could not be obtained. Combined with the lack of a global criterion based on $f_{0}$, this points to the need for a more comprehensive analysis of the signal. For this reason, the remainder of this paper utilizes full-band SAS signals and the AST architecture, which analyzes their time-frequency structure. The AST remained the primary model, while four CNN benchmarks (SqueezeNet, MobileNet, YOLOv8-nano, and ShuffleNetV2 x0.5) used the same full-band log-mel inputs.

### 3.1. Classification Results and Model Comparison

The AST model achieved high and consistent performance across successive runs. Accuracy on the test sets ranged from 0.9444 to 0.9833, and F1 ranged from 0.9432 to 0.9832. The average accuracy was 0.9656±0.0154, precision 0.9750±0.0095, recall 0.9556±0.0261, the F1-score was 0.9651±0.0160, specificity was 0.9756±0.0093, balanced accuracy was 0.9656±0.0154, and ROC-AUC was 0.9953±0.0020. Detailed results are presented in Table 3.

A key element of the evaluation was comparing AST with a baseline model that used only the dominant frequency $f_{0}$. A logistic regression model trained on this single feature, while maintaining the same train/validation/test splits, achieved an accuracy of 0.4733±0.0315, an F1-score of 0.4662±0.0466, and a ROC-AUC of 0.4605±0.0239. This result, which is close to random, confirms that a single frequency does not contain sufficient information for the global classification of recordings from all points. In contrast, AST, which utilizes the full structure of the spectrogram, achieved an accuracy of approximately 96.6% and an ROC-AUC of approximately 0.995.

To provide a broader deep-learning benchmark, Table 4 compares AST with four compact CNN architectures: SqueezeNet, MobileNet, YOLOv8-nano, and ShuffleNetV2 x0.5. All CNNs clearly outperformed the dominant-frequency baseline, with mean accuracies ranging from 0.9381 to 0.9500 and ROC-AUC values from 0.9806 to 0.9912. Among the CNNs, ShuffleNetV2 x0.5 achieved the highest accuracy (0.9500 ± 0.0142), F1-score (0.9507 ± 0.0138), and ROC-AUC (0.9912 ± 0.0035), followed closely by YOLOv8-nano, MobileNet, and SqueezeNet. AST nevertheless provided the highest mean accuracy (0.9656 ± 0.0154), precision (0.9750 ± 0.0095), F1-score (0.9651 ± 0.0160), and ROC-AUC (0.9953 ± 0.0020). ShuffleNetV2 showed slightly higher recall (0.9644 versus 0.9556), but the difference of 0.0088 is small relative to the across-seed variability and is not interpreted as a meaningful advantage without a paired significance test. Overall, the CNN results show that the log-mel representation is highly discriminative, while AST provides the strongest overall balance of metrics in the present experiment.

The ROC curves shown in Figure 12 confirm the model’s high and stable ability to separate classes; the ROC-AUC values for all five runs fall within the range of 0.9925–0.9975. The confusion matrix for a representative run with seed = 53 is shown in Figure 13. The variation in accuracy and F1 scores across seeds is clearly smaller than the difference between AST and the frequency-based model. This indicates that the observed advantage is not due to a single favorable run of the model. At the same time, the results should not be interpreted as evidence of full generalization to new castings: all recordings come from two physical components, and the split was performed at the file level.

### 3.2. Saliency-Based Interpretability Analysis

Figure 14 presents the class-averaged gradient-based saliency maps for correctly classified OK and NOK recordings from the representative seed = 53 run. The OK map shows sensitivity distributed over a broad portion of the patch-covered frequency range, with several localized regions of increased saliency. In contrast, the NOK map exhibits a more pronounced concentration of saliency in the low-frequency region, particularly below approximately 200 Hz, while weaker sensitivity remains present at higher frequencies. The highlighted regions are not confined to a single dominant spectral maximum, which is consistent with the observation that the dominant frequency alone is insufficient for global OK/NOK classification. These maps should be interpreted as model-sensitivity visualizations rather than as evidence that a specific frequency band is a direct physical signature of a material defect.

## 4. Discussion

The frequency-domain results (Table 2, Figure 10) show that internal defects do perturb the SAS self-excitation frequency, but not in a way that a single global threshold can exploit: the sign and magnitude of the shift vary strongly across the six measurement points, from a −68.6% drop at point 2 to a +85.5% rise at point 3. This is consistent with the underlying physics of the method rather than with measurement noise. In a self-excited loop, the system locks onto whichever local vibration mode satisfies the closed-loop gain and phase conditions at the contact point [17]; which mode is selected, and how strongly a given internal inhomogeneity couples to that mode’s strain field, depends on the local mass–stiffness distribution and on the position of the defect relative to the modal nodes and antinodes at that location. A defect located near a node of the locally excited mode will barely perturb $f_{0}$, while the same defect near an antinode of a different, geometry-dependent local mode can shift it substantially.

Heinrich et al. [14] reported a related limitation for classical resonance inspection: frequency shifts due to minor defects can be indistinguishable from shifts caused by ordinary dimensional tolerances once both are of comparable magnitude. Our results extend this observation to a self-excited configuration: although both specimens share the same nominal geometry, part-to-part manufacturing variability cannot be excluded; nevertheless, the observed location dependence demonstrates that a single-frequency criterion is insufficient across the investigated measurement points. This explains, at least qualitatively, the near-chance performance of the $f_{0}$-only baseline (accuracy 0.4733±0.0315, ROC-AUC 0.4605±0.0239): the true class boundary is not a single threshold in one-dimensional frequency space but a point-dependent, and likely non-monotonic, function of it.

A natural engineering response to frequency instability is to constrain the feedback loop to a fixed band, as previously done for anchor diagnostics in mining applications [17,18]. Here this strategy only partially succeeded: the 600–1600 Hz band stabilized self-excitation at points 1 and 4 but not elsewhere, and no single band covered all six locations (Figure 11). This indicates that, unlike the more uniform anchor geometry in [17], the casting under test presents a richer and more spatially heterogeneous set of locally excitable modes, so that restricting the loop bandwidth effectively restricts which points can even sustain self-excitation. Practically, this rules out a simple “one filter fits all measurement points” configuration for this class of parts and motivated the shift to full-band signal acquisition combined with a model expressive enough to handle the resulting mode diversity, rather than trying to engineer that diversity away at the acquisition stage.

The large performance gap between the AST model (accuracy 0.9656±0.0154, ROC-AUC 0.9953±0.0020) and the $f_{0}$-only baseline is informative about what information the dominant frequency discards. A mel-spectrogram retains, beyond the position of the strongest spectral peak, the relative energy of harmonics and side components, the temporal evolution of the self-excited oscillation as it settles into its limit cycle, and fine-grained shifts in secondary spectral content that a single-peak extraction step removes by construction. Local material discontinuities are expected to affect exactly this kind of information: a defect can alter modal damping and hence the shape of the transient onset of self-excitation, redistribute energy toward secondary modes without necessarily shifting the dominant peak, or introduce weak nonlinear components in the vibration response. The self-attention mechanism of the AST, operating jointly over the time and frequency axes of the spectrogram [36,37], is architecturally suited to pick up such distributed, non-localized cues rather than a single scalar feature. This is consistent with the intuition motivating the SSAST line of work [38], where the value of the representation lies in the full time-frequency structure rather than in any single hand-picked descriptor.

It is also notable that a checkpoint pretrained on AudioSet—a general-purpose environmental/audio classification corpus with no obvious relation to structural vibration signals—transferred effectively to this task after fine-tuning. This suggests that the low- and mid-level spectrogram features learned from generic acoustic data (edge/onset detectors, harmonic-structure sensitivity, local time-frequency texture) are sufficiently generic to be reusable for structural diagnostics, which is practically convenient given the modest size of the present dataset (1200 recordings from two physical parts) relative to what training an equivalent architecture from scratch would typically require.

The saliency analysis provides qualitative support for the use of distributed spectro-temporal information: the OK recordings showed a broader distribution of gradient sensitivity, whereas the NOK recordings exhibited a stronger low-frequency concentration. However, saliency measures sensitivity of the model output to the input and does not establish a causal relationship between the highlighted regions and a specific defect mechanism.

The expanded CNN benchmark further qualifies the architecture-specific interpretation. All four CNNs achieved high performance on the same log-mel representation (accuracy 0.9381–0.9500; ROC-AUC 0.9806–0.9912), showing that strong classification is not unique to a transformer. AST retained the highest mean accuracy, precision, F1-score, and ROC-AUC, while ShuffleNetV2 x0.5 was the strongest CNN overall. ShuffleNetV2 showed slightly higher mean recall than AST (0.9644 vs. 0.9556), a difference of 0.0088. This is smaller than the across-seed recall SD for either model (0.0261 and 0.0122, respectively); without a paired significance test, it is not interpreted as a meaningful advantage. Overall, the comparison supports the value of full time-frequency learning more strongly than a large transformer-specific advantage.

### 4.1. Practical Implications for Production-Line Inspection

From an applied standpoint, the results support the two-stage rationale set out in the Introduction. Radiographic and CT-based inspection remain the reference methods for internal defect detection but are ill-suited to 100% inline inspection given cycle times of several minutes against production cycles of the order of tens of seconds [8]. The SAS-based measurement, in contrast, requires roughly 2 s of contact per point (1 s to reach a stable self-excited state plus 1 s of acquisition) and was demonstrated here under robotic automation, which demonstrates technical compatibility in principle with automated inline measurement, although classification performance in such deployment remains to be validated on independent castings.

The frequency-only analysis would not, on its own, justify such deployment, since its diagnostic value is highly location-dependent and requires per-point calibration or thresholding that does not generalize across the part. Within the investigated dataset, the AST-based classifier substantially reduced this dependency by learning directly from the raw time-frequency content, at the cost of requiring a trained deep model and a moderately sized labeled dataset per part family rather than a simple, interpretable threshold.

In an industrial setting this trade-off—a black-box, data-driven classifier versus a transparent but location-sensitive frequency rule—is likely to be the main practical consideration when deciding whether to adopt the method, alongside the need to validate performance on parts beyond the two castings studied here. The broader CNN benchmark likewise shows that compact models can approach AST performance; ShuffleNetV2 x0.5 (343,410 parameters) was the strongest CNN. Inference latency and hardware utilization were not benchmarked, so no deployment-speed advantage is claimed.

### 4.2. Limitations and Threats to Validity

Several limitations qualify the strength of the conclusions that can be drawn. The most significant is that only two physical castings were available (one OK, one NOK), and the train/validation/test split was performed at the recording level rather than at the casting level. Because multiple recordings originate from the same two physical parts, the reported metrics should be interpreted as a measure of how well the model separates the acoustic signatures of these particulardefect-free and defective regions, rather than as an estimate of generalization to previously unseen castings; the model may in part be learning part-specific acoustic fingerprints that do not transfer. This is a limitation concerning generalization rather than the model’s underlying ability to detect acoustic differences associated with local defects: the accuracy, F1-score, and ROC-AUC values reported above confirm high classification performance under the conditions of the present experiment.

A leave-one-casting-out or, more realistically, a multi-casting validation design would be required to disentangle genuine defect-related discriminative power from casting-specific idiosyncrasies; in future studies we plan to expand the dataset to a larger number of physical parts and to apply group validation at the casting level.

Second, the classification problem was posed as strictly binary (OK/NOK) and does not distinguish between defect types (porosity versus non-metallic inclusions) or defect severity, both of which are of practical interest for triage decisions on a production line. The result should therefore be interpreted as a classification of the local material state based on the SAS signal, rather than as a classification of defect type; extending the model to multi-class defect identification is a natural direction for further research.

Third, the AST classification pipeline used only the unfiltered, full-band recordings; it remains open whether combining the (partially informative) filtered-band measurements with the AST representation, e.g., as additional input channels, would improve robustness or merely add redundant information given the band-selection issues discussed above.

Finally, the added gradient-based saliency analysis provides an initial qualitative indication of the time-frequency regions to which the AST predictions are most sensitive. Nevertheless, saliency does not establish a causal physical relationship between the highlighted regions and a specific defect mechanism, and the two-casting design prevents separating defect-related cues from all possible casting-specific acoustic characteristics.

## 5. Conclusions

The conducted research has shown that the SAS self-excited acoustic system enables the repeatable recording of a casting’s local dynamic response and can serve as a source of information useful in the nondestructive diagnosis of local material inhomogeneities. Measurements taken without feedback loop filtering revealed clear differences between the responses of the OK and NOK castings; however, the magnitude and direction of the change in the dominant frequency depended on the measurement point. This means that a single self-excitation frequency value does not allow for the definition of a single global decision criterion for all analyzed locations. The use of bandpass filtering limited frequency jumps and stabilized the system’s operation within the selected range; however, none of the tested bands—600–1600 Hz, 1600–2600 Hz, and 2600–3600 Hz—simultaneously provided a stable response at all six measurement points. This confirms that a single fixed filtering band cannot be treated as a universal setting for the various local inhomogeneities of the casting under examination. The introduction of full-band SAS signal analysis using the Audio Spectrogram Transformer (AST) architecture made it possible to utilize the information contained in the entire time-frequency representation of the signal, rather than solely at the location of the dominant spectral maximum. The AST model achieved an average accuracy of 0.9656±0.0154, an F1-score of 0.9651±0.0160, and a ROC-AUC of 0.9953±0.0020, whereas the baseline model, which used only the dominant frequency, achieved an accuracy of 0.4733±0.0315 and a ROC-AUC of 0.4605±0.0239.

Such a large difference between the results of the two approaches indicates that the features relevant for distinguishing between local OK and NOK states are contained within the broader structure of the SAS signal and can be effectively extracted by the AST model.

The obtained results support the feasibility of combining a self-excited acoustic system with deep learning methods for classifying local material states within the investigated experimental setup. Accordingly, the present study should be regarded as a proof-of-concept for within-experiment defect-state classification rather than as a casting-independent validation of the proposed classifier. All four compact CNN baselines also achieved high performance on the same log-mel representation, with ShuffleNetV2 x0.5 the strongest CNN. AST retained the highest mean accuracy, precision, F1-score, and ROC-AUC. Thus, the main gain came from moving beyond a single dominant-frequency descriptor; the additional transformer-specific gain was comparatively modest in this experiment.

The immediate priority for future work is scaling the dataset to a larger and more diverse population of physical castings, with validation strictly stratified at the casting level, to obtain a defensible estimate of generalization to unseen parts. A second direction is extending the classification target from a binary OK/NOK label toward defect type and/or severity estimation, which would require X-ray-derived ground truth beyond simple pass/fail labeling. Third, the present gradient-based saliency analysis provides an initial qualitative view of the time-frequency regions contributing to AST decisions; future work should extend this analysis using complementary interpretability methods and independent castings to determine whether the observed patterns remain stable across parts and can be linked to specific defect mechanisms. Finally, given the partial diagnostic value observed for filtered-band measurements, a hybrid approach combining band-limited measurements at points where a stable, informative band exists with full-band AST analysis elsewhere may offer a practical middle ground between interpretability and robustness in an eventual industrial implementation.

## Figures and Tables

**Figure 1:**
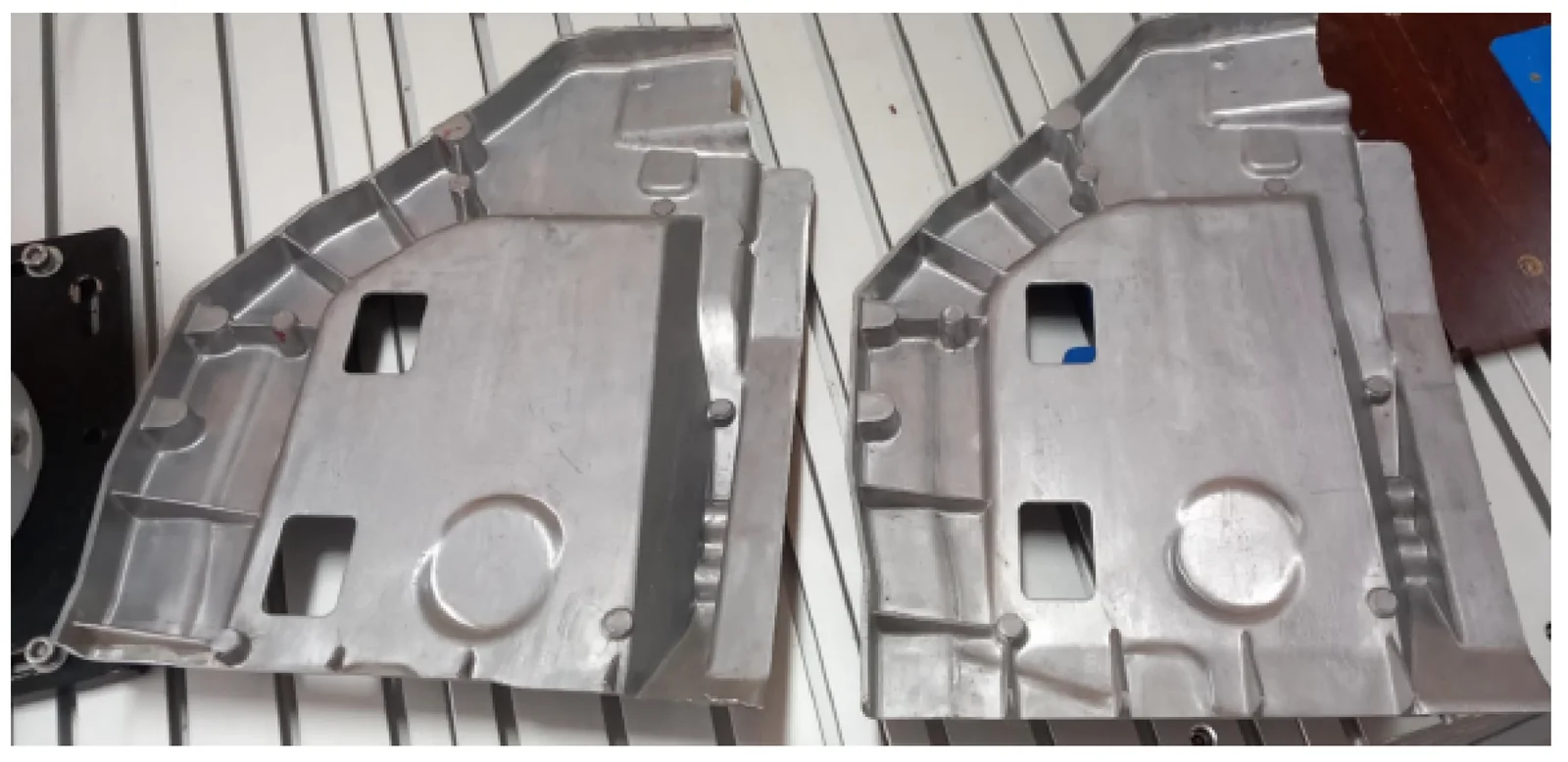
Aluminum alloy passenger car suspension component used in the experimental study.

**Figure 2:**
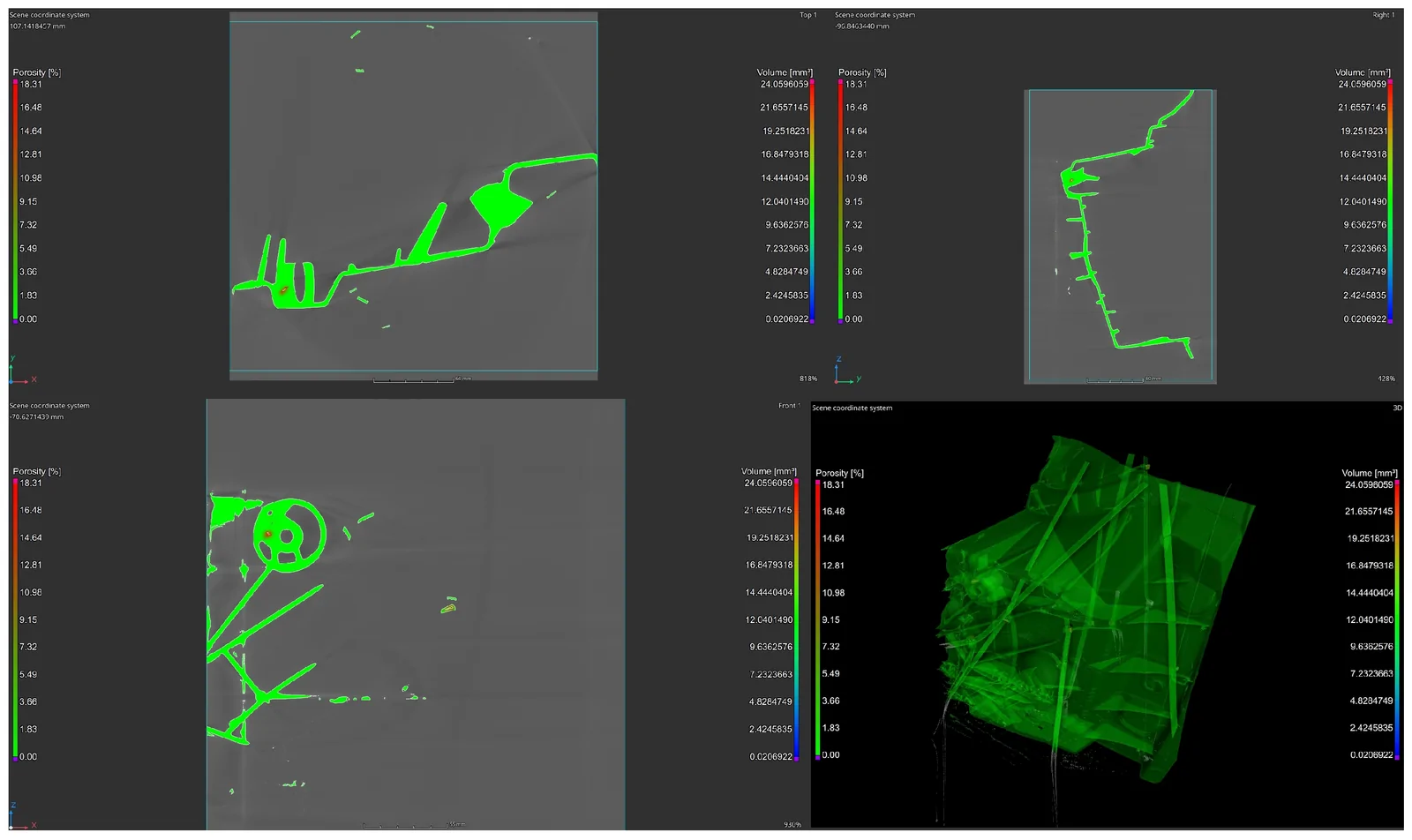
Results from the X-ray examination of the NOK sample. View of an inclusion in the material at a selected measurement point.

**Figure 3:**
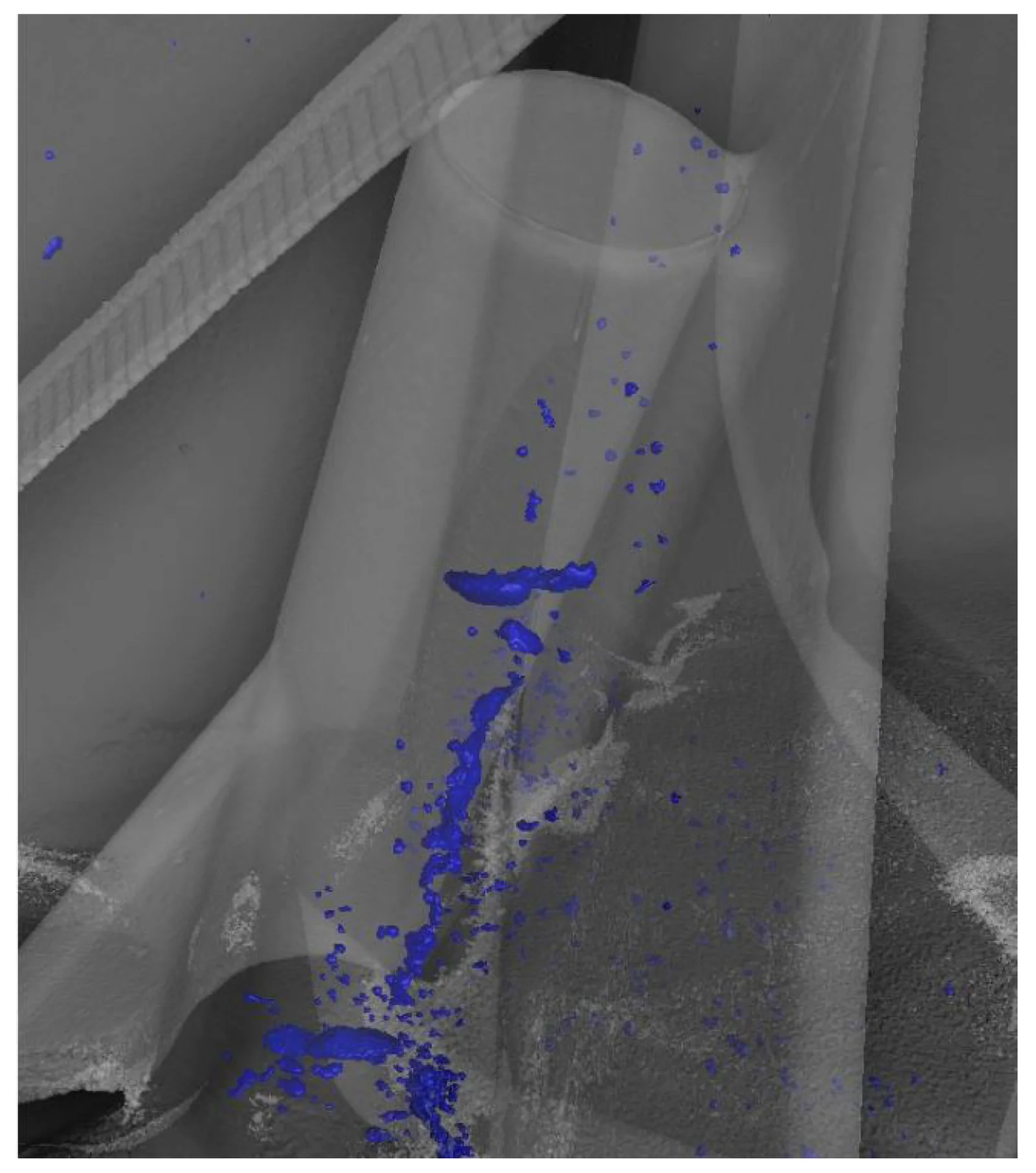
Example injection region of a casting, X-ray CT volume rendering. The casting surface is rendered as a semi-transparent gray body, and the detected closed-air pores are colored blue.

**Figure 4:**
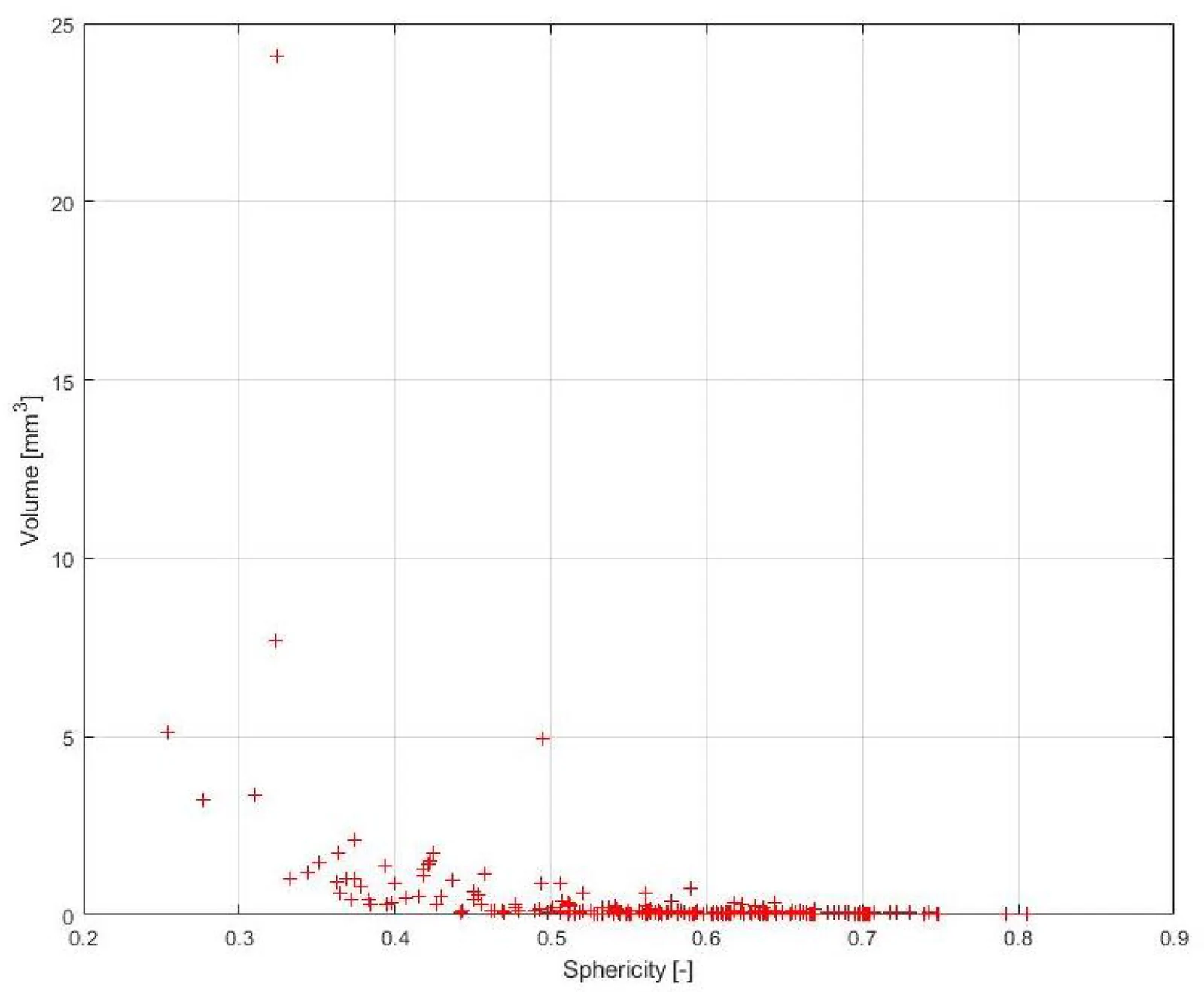
Porevolume versus sphericity for casting S1 (n=417 pores), linear axes. Each cross represents one pore.

**Figure 5:**
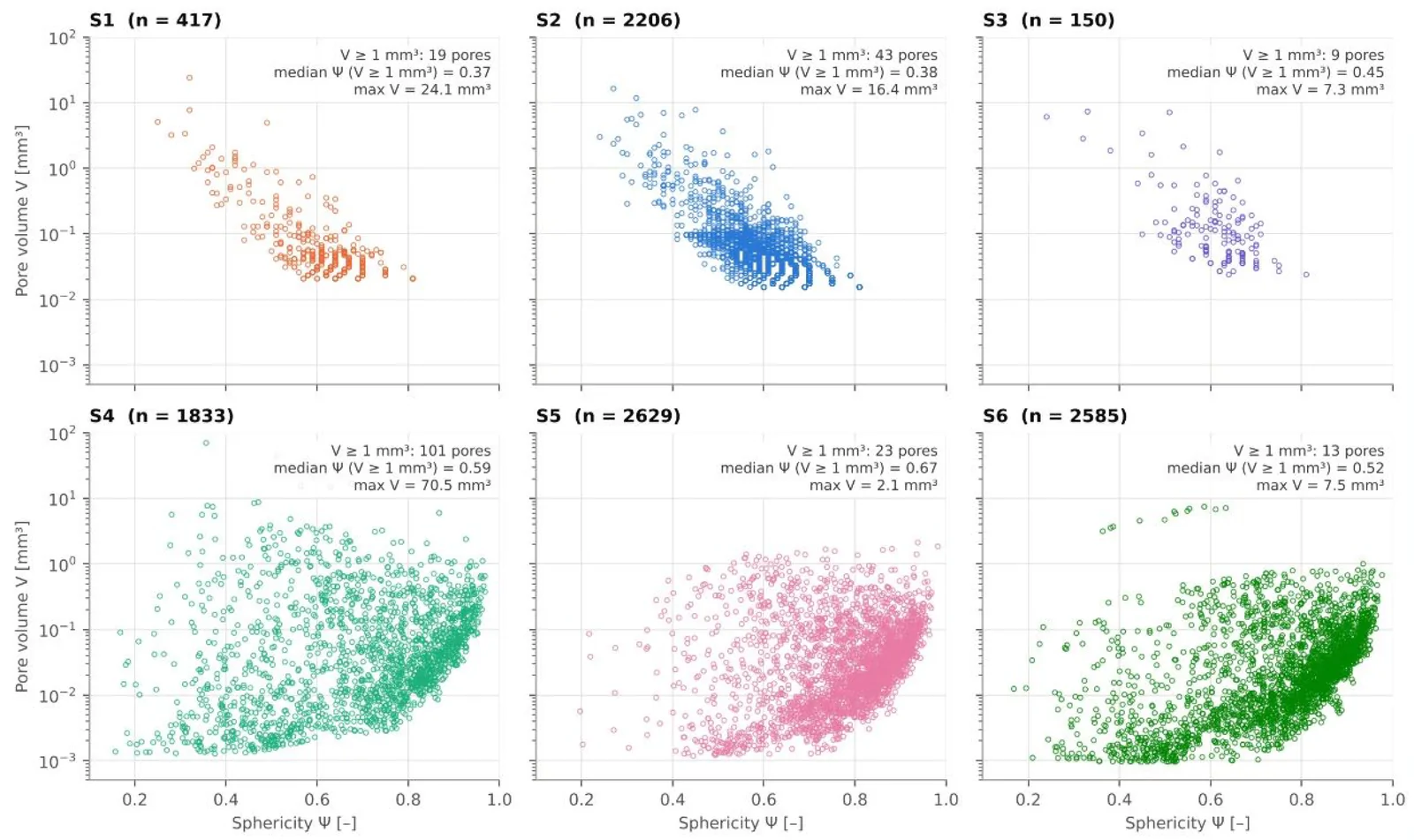
Pore volume (logarithmic axis) versus sphericity for castings S1–S6, plotted on common axes. Each circle represents one pore. The annotations give the number of pores of at least 1 mm^3^, the median sphericity of those pores, and the volume of the largest pore.

**Figure 6:**
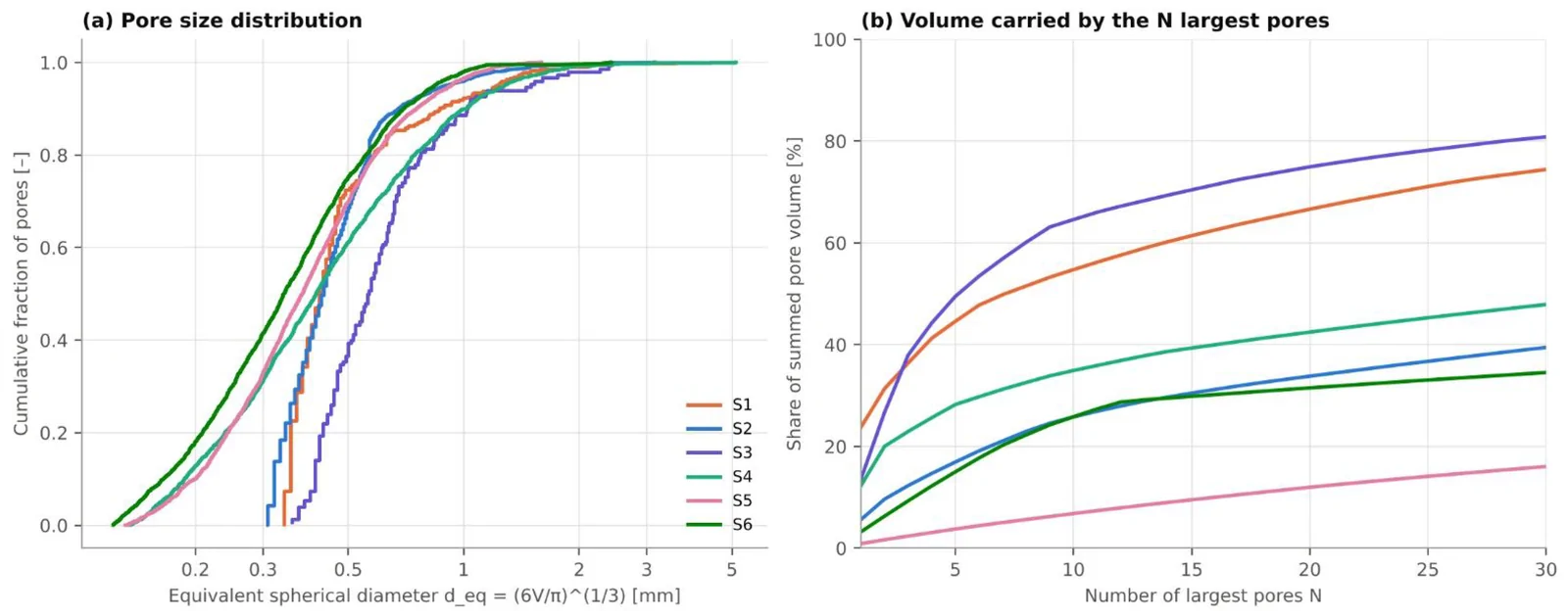
(**a**) Empirical cumulative distribution of the equivalent spherical diameter $d_{\mathrm{eq}}$ of pores in castings S1–S6 (logarithmic axis). (**b**) Share of the summed pore volume carried by the *N* largest pores; colors as in (**a**).

**Figure 7:**
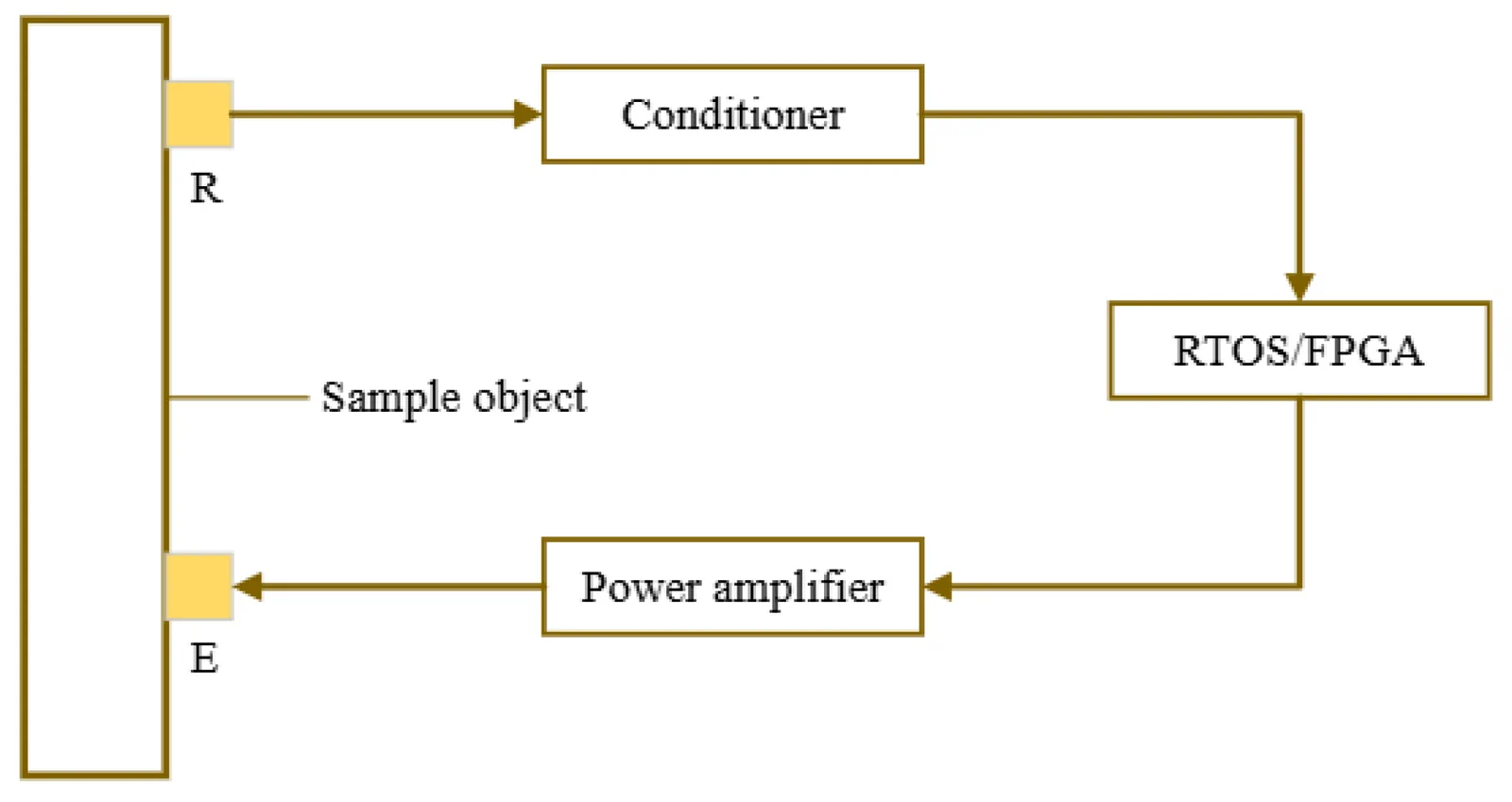
Schematic diagram of a self-excited acoustic system (SAS): E—vibration exciter, R—sensor (accelerometer).

**Figure 8:**
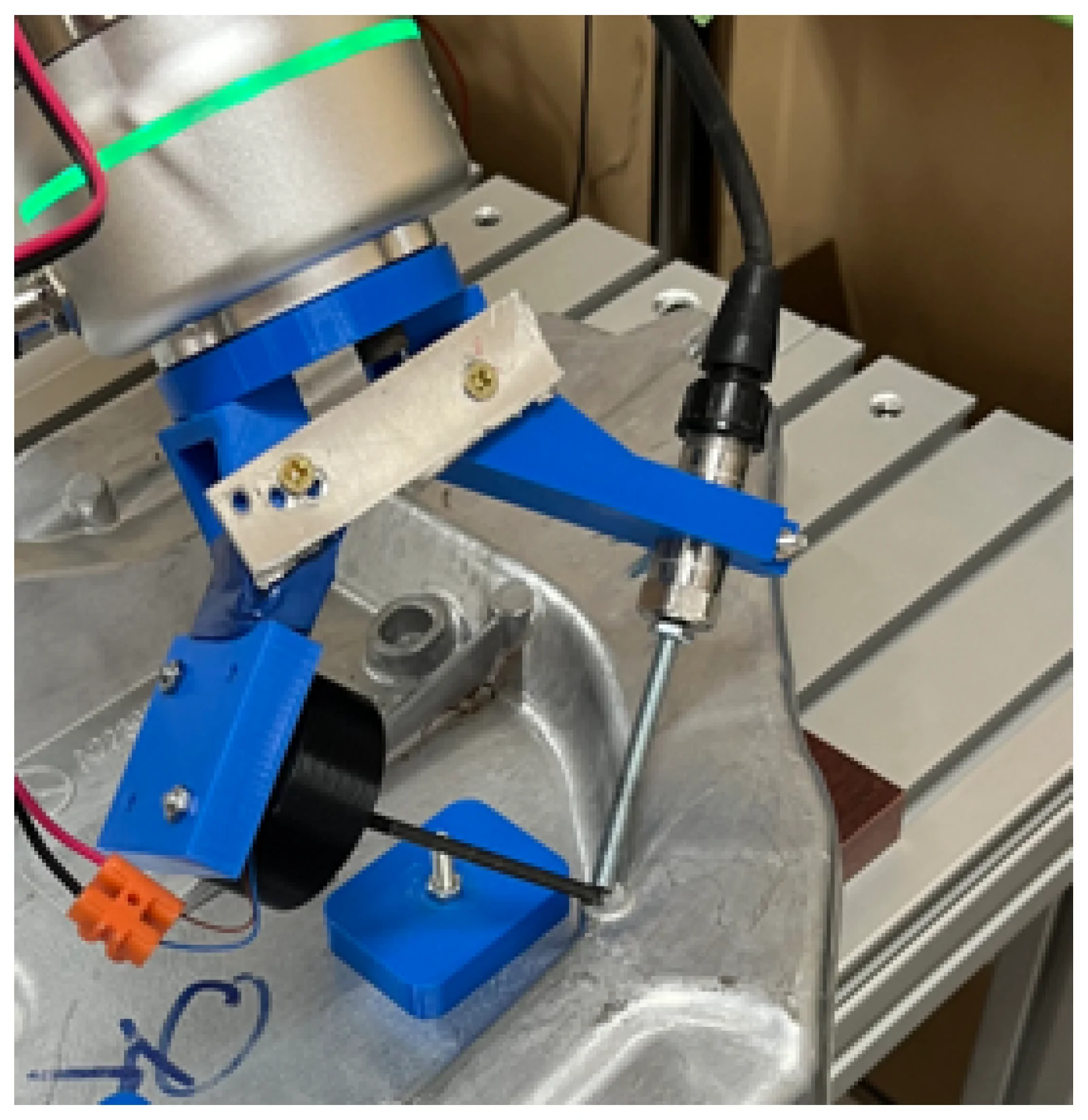
The SAS system’s measuring head mounted on the end of a robotic arm while measuring an aluminum casting.

**Figure 9:**
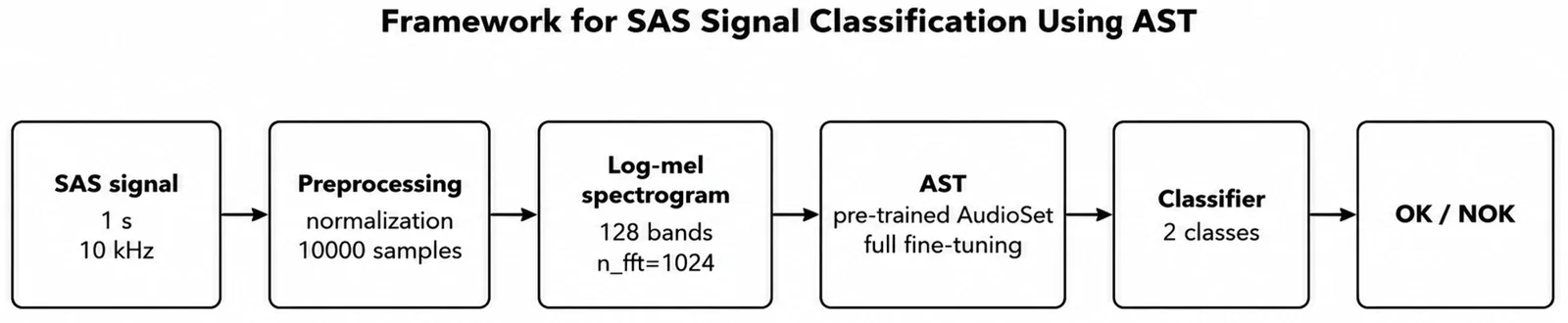
SAS signal classification framework using AST architecture.

**Figure 10:**
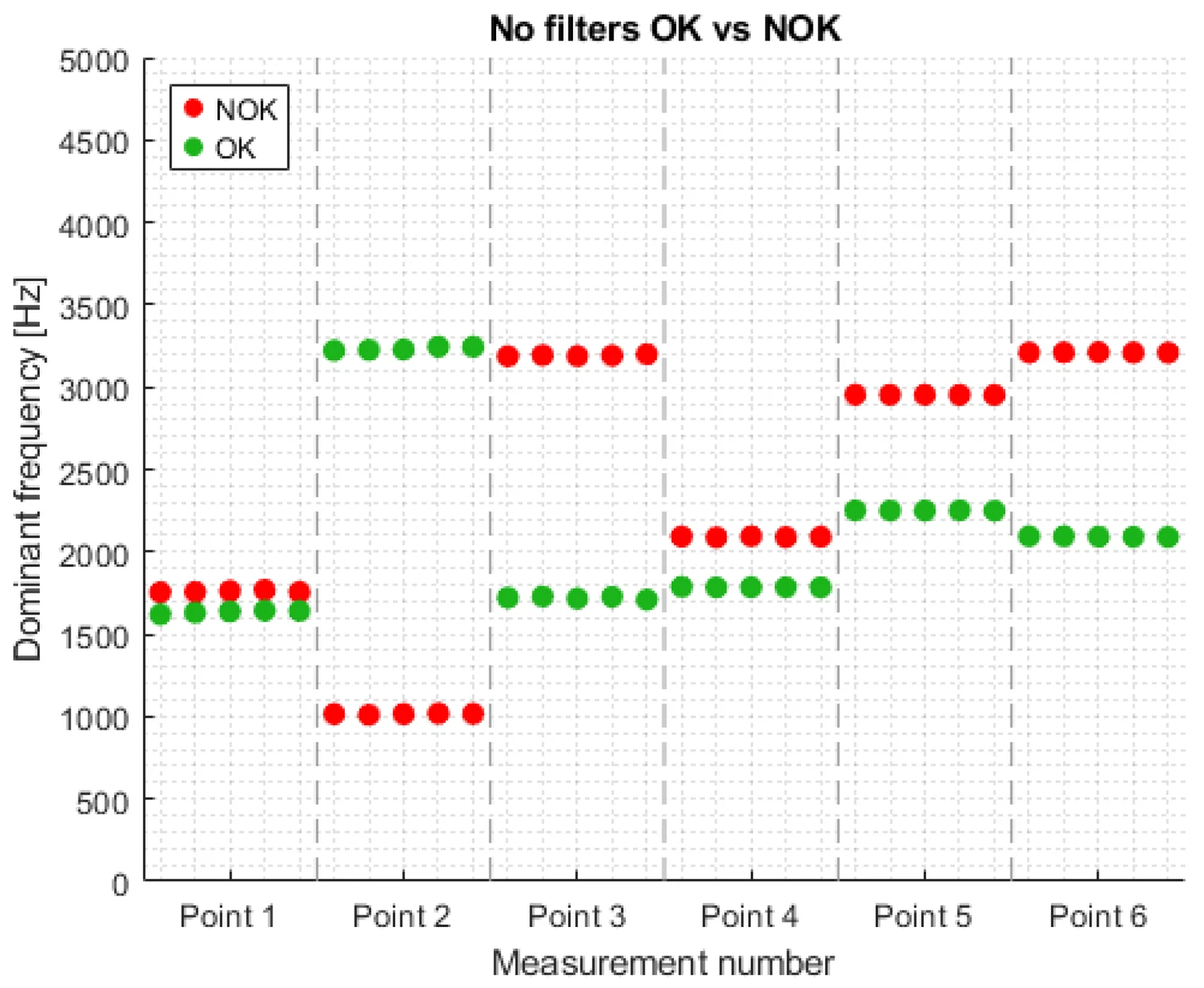
Dominant frequency values for the OK and NOK samples at six measurement points without feedback loop filtering.

**Figure 11:**
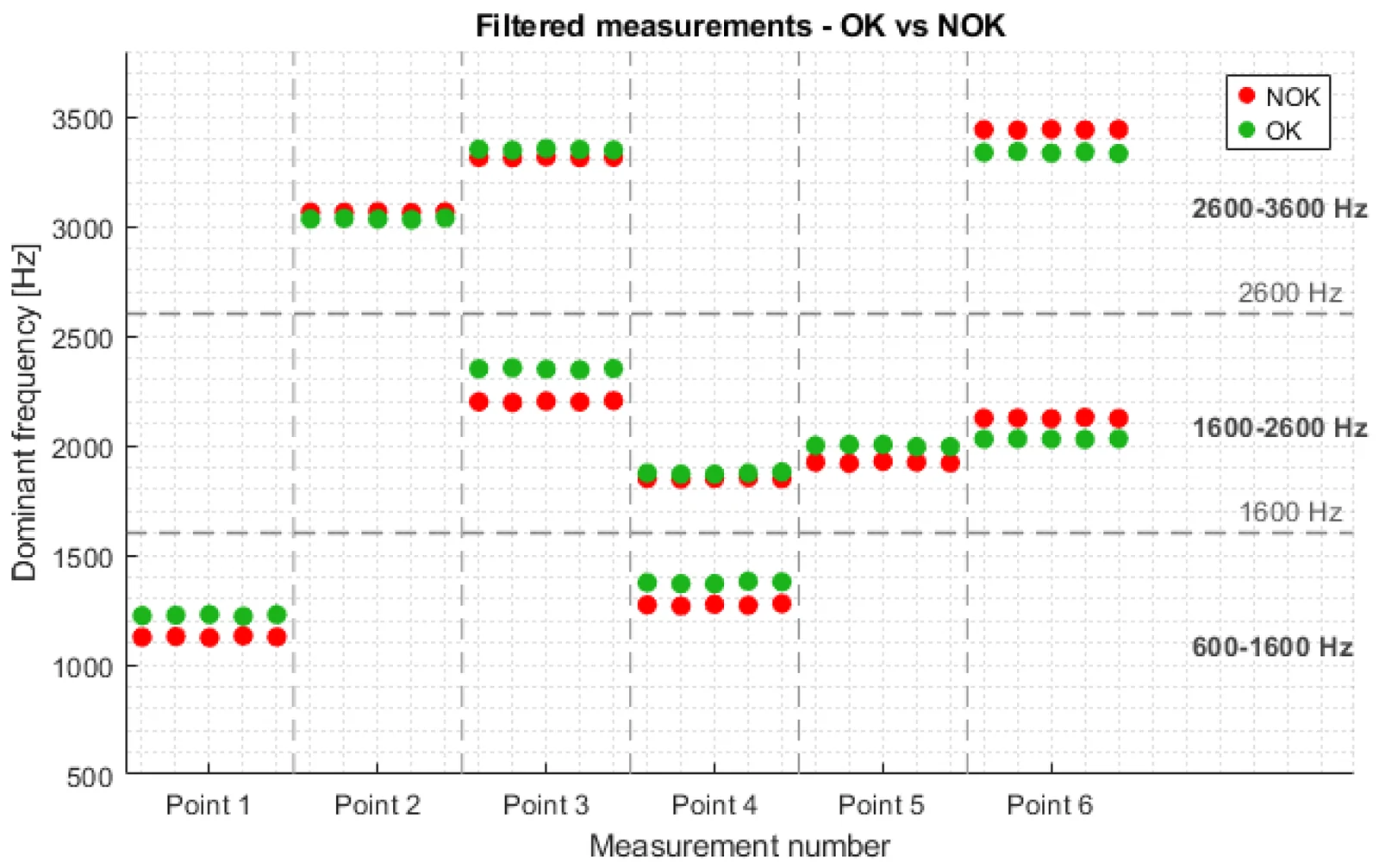
Results of self-excitation frequency measurements with bandpass filtering of the feedback loop.

**Figure 12:**
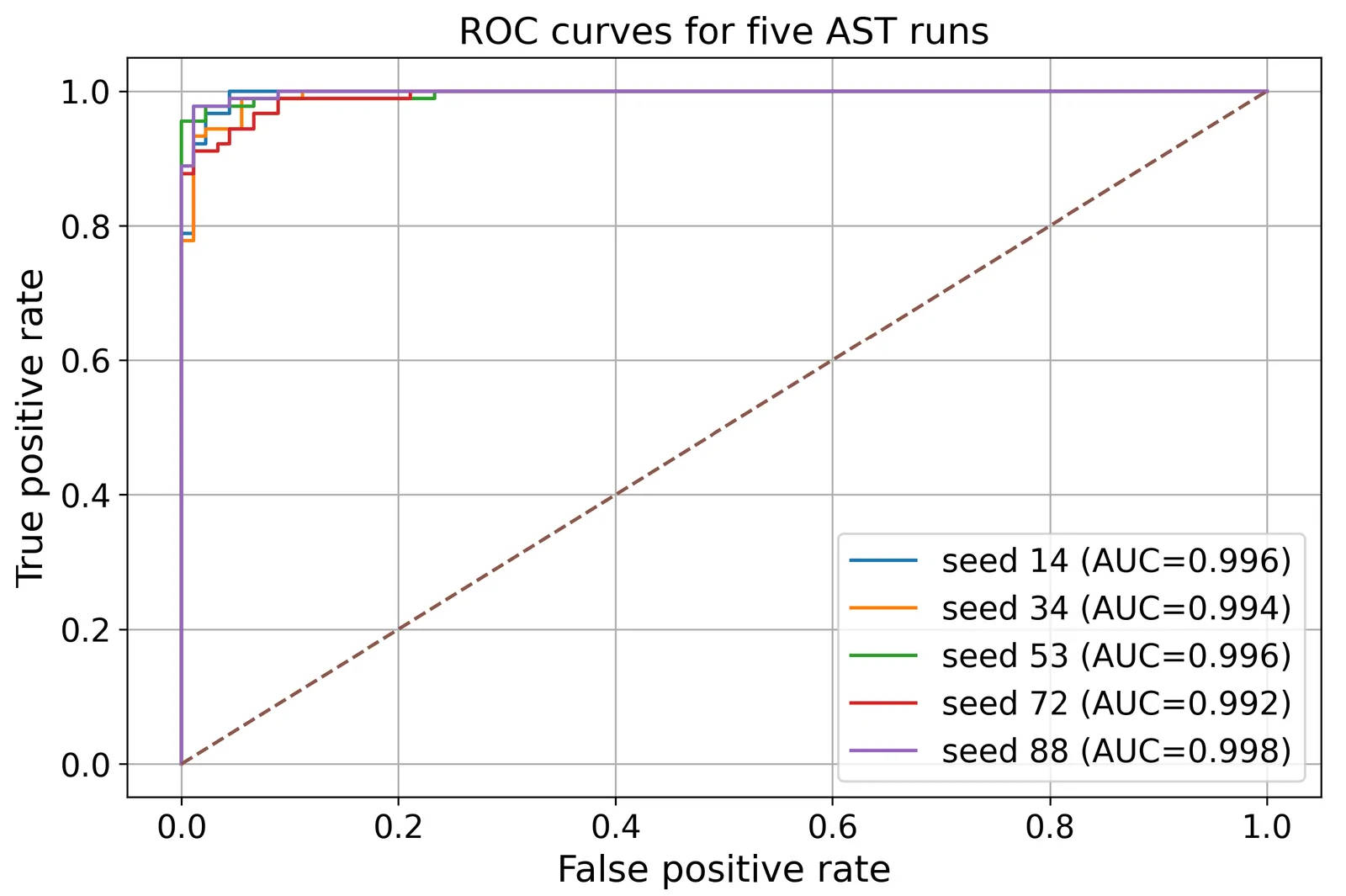
ROC curves for five independent runs of the AST model.

**Figure 13:**
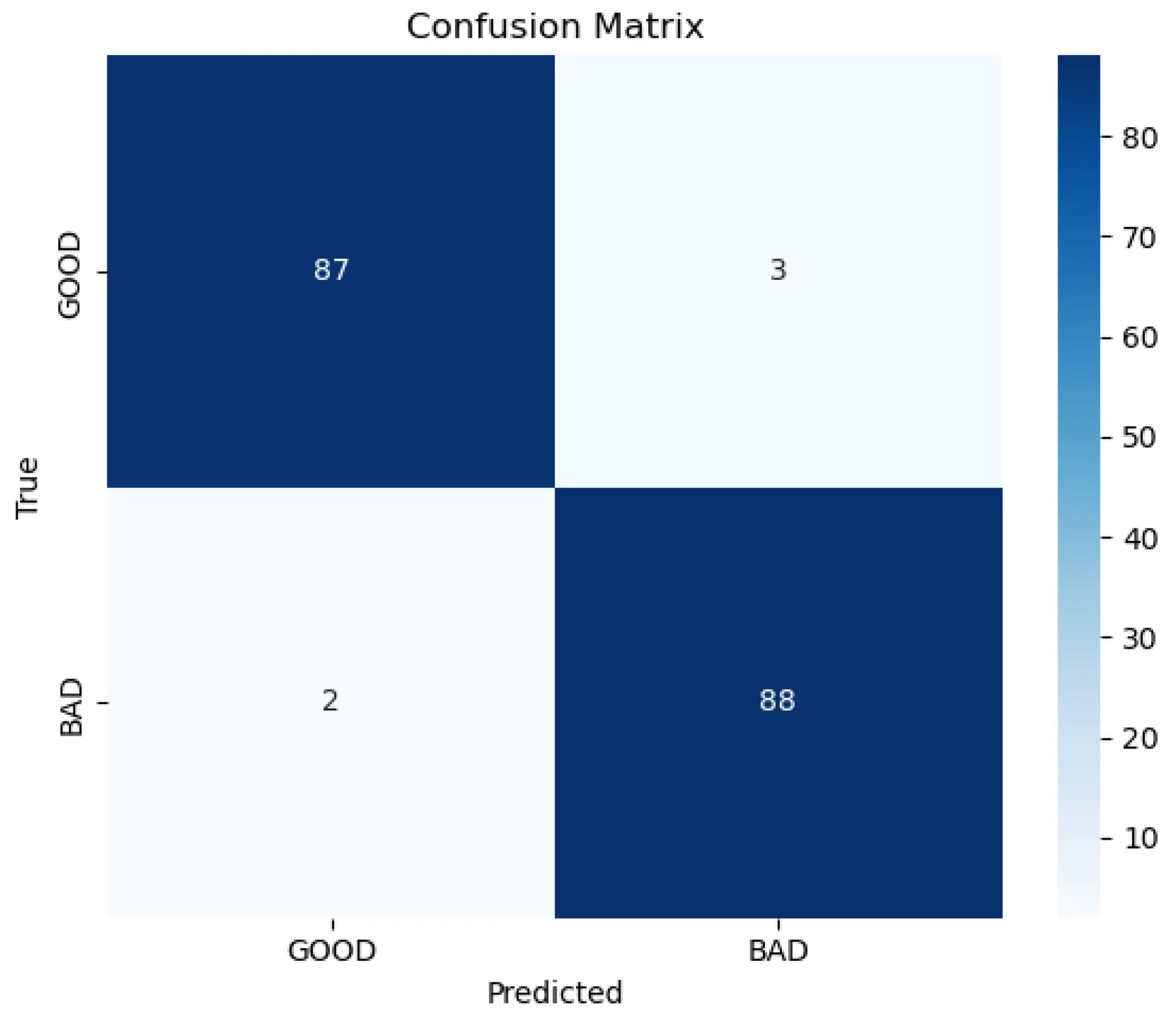
Confusion matrix for a representative run with seed = 53 (accuracy = 0.9722).

**Figure 14:**
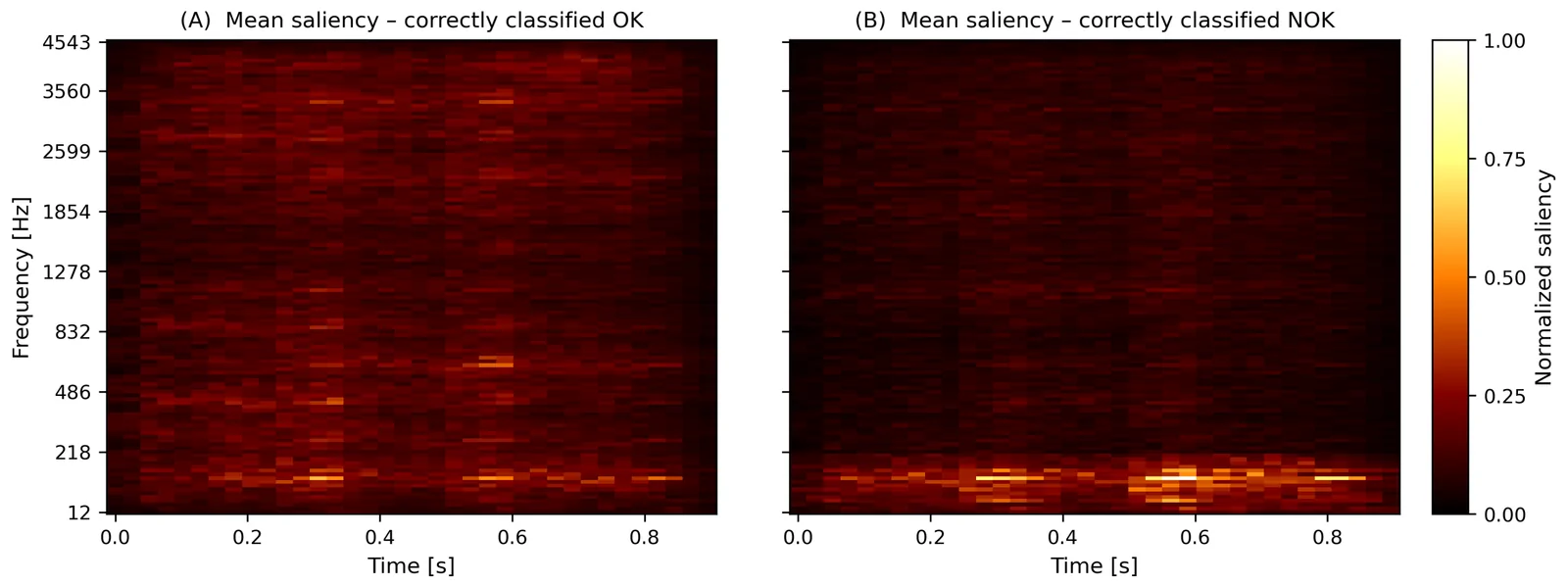
Mean gradient-based saliency maps for correctly classified test recordings obtained with the AST model trained with seed = 53: (**A**) OK class (*n* = 87) and (**B**) NOK class (*n* = 88). Both maps were jointly normalized to [0, 1] using a common scale.

**Table 1:** Pore statistics for the individual castings.

Casting	*N*	Porosity (%)	*V* Med. (mm^3^)	*V* P99 (mm^3^)	*V* Max (mm^3^)	$d_{\mathrm{eq}}$ Max (mm)	$D_{\mathrm{circ}}$ Max (mm)	Ψ Med.	*N* (V≥1 mm^3^)	Top-10 Share (%)
S1	417	0.012	0.039	3.35	24.1	3.58	11.2	0.62	19	55
S2	2206	0.039	0.042	1.71	16.4	3.15	8.8	0.60	43	26
S3	150	0.016	0.094	6.60	7.3	2.41	9.5	0.62	9	65
S4	1833	0.071	0.037	3.42	70.5	5.13	26.7	0.74	101	35
S5	2629	0.025	0.029	0.92	2.1	1.60	6.1	0.80	23	7
S6	2585	0.031	0.021	0.68	7.5	2.43	12.2	0.79	13	26

Porosity is the summed volume of the detected pores (ΣV) related to the casting volume; Top-10 share is the share of ΣV carried by the ten largest pores. *V* P99 is the 99th percentile of pore volume; $d_{\mathrm{eq}}={\left(6V/\pi \right)}^{1/3}$ is the equivalent spherical diameter; $D_{\mathrm{circ}}$ is the diameter of the circle circumscribed on the pore; Ψ is the sphericity. In S1–S3, the sphericity does not exceed 0.81, and in S4–S6 it reaches 0.97, so its values should be compared only within these groups.

**Table 2:** Self-excited vibration frequencies at six points on both specimens (OK and NOK) across the full range of the feedback loop.

Measurement Point	OK (Hz)	NOK (Hz)	Change (%)
1	1635±14	1760±9	+7.6%
2	3233±12	1016±5	−68.6%
3	1721±11	3192±8	+85.5%
4	1786±2	2091±4	+17.1%
5	2251±1	2953±1	+31.2%
6	2090±3	3210±2	+53.4%

**Table 3:** AST classification results for five runs, along with the mean ± standard deviation.

Seed	Accuracy	Precision	Recall	F1	Specificity	ROC-AUC
88	0.9833	0.9888	0.9778	0.9832	0.9889	0.9975
14	0.9722	0.9775	0.9667	0.9721	0.9778	0.9960
53	0.9722	0.9670	0.9778	0.9724	0.9667	0.9962
34	0.9556	0.9767	0.9333	0.9545	0.9778	0.9943
72	0.9444	0.9651	0.9222	0.9432	0.9667	0.9925
Mean ± SD	0.9656±0.0154	0.9750±0.0095	0.9556±0.0261	0.9651±0.0160	0.9756±0.0093	0.9953±0.0020

**Table 4:** Comparison of the dominant-frequency baseline, CNN architectures, and the AST model.

Model	Accuracy	Precision	Recall	F1	ROC-AUC
Dominant frequency $f_{0}$	0.4733±0.0315	0.4716±0.0336	0.4622±0.0627	0.4662±0.0466	0.4605±0.0239
CNN- SqueezeNet	0.9381±0.0170	0.9345±0.0191	0.9416±0.0203	0.9380±0.0180	0.9806±0.0056
CNN-MobileNet	0.9430±0.0157	0.9410±0.0178	0.9470±0.0170	0.9440±0.0166	0.9847±0.0045
CNN-YOLOv8-nano	0.9470±0.0148	0.9441±0.0162	0.9507±0.0148	0.9474±0.0151	0.9884±0.0039
CNN-ShuffleNetV2 x0.5	0.9500±0.0142	0.9375±0.0172	0.9644±0.0122	0.9507±0.0138	0.9912±0.0035
AST	0.9656±0.0154	0.9750±0.0095	0.9556±0.0261	0.9651±0.0160	0.9953±0.0020

## Data Availability

The original data presented in the study are openly available in repository AST_inclusion at https://github.com/pgut-agh/AST_inclusion, accessed on 23 October 2026.
